# High-Throughput Microbial Community Analyses to Establish a Natural Fungal and Bacterial Consortium from Sewage Sludge Enriched with Three Pharmaceutical Compounds

**DOI:** 10.3390/jof8070668

**Published:** 2022-06-25

**Authors:** Alejandro Ledezma-Villanueva, Tatiana Robledo-Mahón, Cinta Gómez-Silván, Gabriela Angeles-De Paz, Clementina Pozo, Maximino Manzanera, Concepción Calvo, Elisabet Aranda

**Affiliations:** 1 Institute of Water Research, University of Granada, Ramón y Cajal, 4, 18071 Granada, Spain; ledezmavilla@gmail.com (A.L.-V.); depazang.gab@gmail.com (G.A.-D.P.); clpozo@ugr.es (C.P.); manzanera@ugr.es (M.M.); ccalvo@ugr.es (C.C.); earanda@ugr.es (E.A.); 2 Department of Microbiology, University of Granada, 18071 Granada, Spain; 3 Department of Environmental Science, Policy, and Management (ESPM), University of California, Berkeley, CA 94710, USA; c.gomez.silvan@gmail.com; 4 Environmental Genomics and System Biology (EGSB), Lawrence Berkeley National Laboratory, 717 Potter St., Bld. 977, Berkeley, CA 94710, USA

**Keywords:** sewage sludge, emerging pollutants, selective pressure, microbial communities, illumina MiSeq

## Abstract

Emerging and unregulated contaminants end up in soils via stabilized/composted sewage sludges, paired with possible risks associated with the development of microbial resistance to antimicrobial agents or an imbalance in the microbial communities. An enrichment experiment was performed, fortifying the sewage sludge with carbamazepine, ketoprofen and diclofenac as model compounds, with the aim to obtain strains with the capability to transform these pollutants. Culturable microorganisms were obtained at the end of the experiment. Among fungi, *Cladosporium cladosporioides*, *Alternaria alternata* and *Penicillium raistrickii* showed remarkable degradation rates. Population shifts in bacterial and fungal communities were also studied during the selective pressure using Illumina MiSeq. These analyses showed a predominance of Ascomycota (*Dothideomycetes* and *Aspergillaceae*) and *Actinobacteria* and *Proteobacteria*, suggesting the possibility of selecting native microorganisms to carry out bioremediation processes using tailored techniques.

## 1. Introduction

Many different technologies have been developed to guarantee the efficient municipal and industrial wastewater recycling of an increasing population, improving the capacity and efficiency of wastewater treatment plants (WWTPs) [1,2]. Currently, the accuracy of technology in detecting chemicals at trace levels is crucial in identifying emerging contaminants (ECs) in sewage sludge. ECs have been detected in primary and secondary sewage sludge after sewage sludge treatment by anaerobic digestion, and even at higher concentrations due to concentration processes. Included in this variety of compounds are novel and old types of contaminants, human and veterinary drugs, nanomaterials, personal care products, paints and coatings, among several others [3,4]. Amid these chemical components, pharmaceutical active compounds (PhACs) represent one of the most important groups, since they can exert changes in microbial populations. They can also promote resistance to drugs in potential pathogen microorganisms, with enormous consequences to human health [5,6]. Hormones and non-steroidal anti-inflammatory drugs (NSAIDs) represent two of the main groups of contaminants with a proven potential risk for environmental organisms and humans, some of which have recently been included in the priority list of pollutants. NSAIDs, such as diclofenac and ketoprofen, and antiepileptic drugs, such as carbamazepine, have been frequently detected in wastewater, sewage sludge and soils [7]. In activated sludge, these pollutants endure the biodegradation process because of their nature, low concentration and retention status in the matrix of the sludge. These remain untreated and in continuous accumulation [8,9]. Deep concern for the possible harmful effects from long term exposure to these organic compounds has started to grow in society, as these molecules have been detected in WWTPs, air, soil and water of clean environments. The sewage sludge from WWTs is usually stabilized in anaerobic digestion to obtain energy, and, after that, the dried residue is further composted for its application as a soil amendment [10]. Even though these compounds may be completely or partially removed in WWTPs, the recalcitrant and bioaccumulative properties of some of these pollutants enable their entrance in the food chain [11]. Thus, the development of new strategies and technologies are needed to deal with the dispersion of these compounds into the environment via sewage sludge. 

Physical and chemical treatments have been considered for their high efficiency in the removal of ECs from WWTPs effluents [12]. Nonetheless, biological treatments have been widely preferred and applied for the removal of ECs. This process involves biochemical transformations in which ECs are degraded by microorganisms such as algae, bacteria, and fungi into smaller molecules [13]. Microorganisms use organic compounds as primary substrates for their cell growth, inducing enzymes for their assimilation [14] and, finally, these molecules can be mineralized into carbon dioxide and water. 

At present, information on the diversity of microorganisms and enzymes with the capacity to degrade ECs is quite limited. Therefore, future studies must be carried out to identify the microorganisms acting on one of the most dominant new pollutants, exploring in further depth to what extent autochthonous microorganisms can degrade ECs in the environment. As sewage sludge is one of the main sources of ECs and a rich environment of adapted microorganisms, this residue could constitute a source of ECs-degraders. Previous studies have been performed using sewage sludge to obtain microorganisms with biodegradation capabilities. For instance, selective pressure has been applied to the study of antibiotic resistance genes (ARGs) [15]. Furthermore, other studies have shown an increment in diversity under non-lethal selective pressure of the target pollutant (e.g., Bisphenol-A) in addition to carbon source. These processes can promote the growth of microorganisms and their adaptation to the concentration of contaminants and can contribute to the establishment of cooperative relationships that improve the degradation of the target compound [16,17]. The treatment of sewage sludge by composting is widely applied to revalorize this residue. Moreover, composting is an optimal ecosystem in which microorganisms with different metabolic capabilities can be found. Previous studies have shown effectiveness in degrading triclosan and antibiotics in sewage sludge composting by native microorganisms [18,19]. 

In addition to this, combining high-throughput sequencing technologies and isolation strategies has led to a greater understanding of the microbiome that inhabits polluted environments, underlying organismal rarity, metabolism profile and its relevance in their own ecology [20,21,22]. Even though there is a level of specialization between the decomposers from the microbial communities, information about specialist taxa that degrade specific compounds is limited. In addition, limited information is available about the dominance of specialist taxa that perform the degradation of compounds of a specific chemical composition. 

In this study, the occurrence of ECs in undigested and digested sewage sludge was determined. Additionally, the genomic profile of native fungi and bacteria in both residues were studied during a selective pressure experiment with carbamazepine, ketoprofen and diclofenac. Finally, microorganisms were isolated to discover the degradation capabilities for further native degrader consortium construction to be used in further biotechnological processes.

## 2. Materials and Methods

### 2.1. Sample Collection

Sewage sludge was collected in the WWTPs “Los Vados” (coordinates in decimal degrees: 37.19121, −3.67639) Granada (Spain) in August 2018. Two types of sewage sludge were used: raw sewage sludge before anaerobic digestion (rSS) and sewage sludge after the anaerobic digestion process, digested sludge (dSS). Samples were disposed following biosafety protocols inside amber sterile glass bottles (~2 L) and transported to the lab in containers with ice. Half of each sample was taken to immediately begin the selective pressure experiments. The other half was stored at −20 °C for DNA extraction and for lyophilization to perform ECs analytical analysis. Bulking samples (B) were provided by the biosolid plant Biomasa del Guadalquivir S.A (Granada, Spain), composed of pruning residues. These are conventionally used in the composting system carried out in the same biosolid plant for the sewage sludge composting processes [23]. 

### 2.2. Selective Pressure Experiments

#### 2.2.1. Setting the Flask Content for Selective Pressure Experiments

A bulking agent, which contributed to the composition of the microbiota [24], was included in addition to rSS and dSS. This was carried out to approximate the conditions of the composting process in the selective pressure experiments. For this, a quantity of 9 g of rSS and dSS samples were mixed with 1 g of B (previously grinded to obtain a homogeneous particle size) in sterilized Erlenmeyer flasks. They were filled with 60 mL of a modified Kirk and Bushnell Haas (BH) media [25,26] (Tables S1 and S2) to analyze the differences between fungal and bacterial growth. The selective media Kirk and BH were used for stimulating the actual communities of fungi and bacteria, respectively; they had also been selected in previous research projects focusing on the degradation and transformation of xenobiotics [27,28]. Carbamazepine (CBZ), ketoprofen (KET) and diclofenac (DCF), all purchased from Sigma Aldrich (St. Louis, MO, USA. ≥98% purity), were added to each flask to reach a final concentration of 50 μM for each compound. For each condition, three flasks were prepared to perform the analysis in triplicate, plus an additional control flask before incubation, i.e., time = 0 (DNA isolation, see Section 2.3) (Figure S1). The prepared flasks (rSS+B in Kirk, rSS+B in BH, dSS in Kirk and dSS in BH) were incubated for 7 days at 28 °C and 120 rpm to start the selective pressure experiment. 

#### 2.2.2. Setting the Pharmaceutical Pressure of the Flask Experiment

After 1 week of incubation, a weekly transfer was performed, transferring 10 mL of each flask into 50 mL of appropriate fresh media. To create the conditions for the fungal community, a booster of the three compounds was added weekly. At the beginning and at the end of each week-old flask, an aliquot of media was taken and immediately centrifuged twice at 14,000 rpm for 5 min. The supernatant was transferred into an HPLC glass vial and stored at -20 °C for further analysis to determine the PhACs concentration (Section 2.5). This transfer was repeated each week for a total of 9 weeks with the predecessor flask. During weeks 0, 5 and 8, a sample of 2 mL of the contents of the flask was frozen at −20 °C for total genomic DNA extraction.

### 2.3. Analysis of Fungal and Bacterial Communities by Illumina MiSeq Sequencing

#### 2.3.1. Sample Preparation and DNA Isolation

To analyze the microbial communities based on DNA analysis, total genomic DNA extraction of the starting materials B, rSS and dSS, as well as from the selective pressure experiments (flasks after 0, 5 and 8 weeks of selective pressure), was performed. For that, a pre-treatment was performed for solid samples (rSS, dSS and B), adding a 0.9% saline solution in a ratio 1:3 (*w*/*v*), sonicated for 10 min and centrifuged twice 800 rpm for 10 min to remove the water phase. The bottom part was subsequently centrifuged at a pulse of 1 min/14,000 rpm to obtain a concentrated pellet. For liquid samples (flask content), the total volume was centrifuged at 5000 rpm for 10 min. The biomass was recovered with a 0.9% saline solution and sonicated for 10 min and continued as described before. A pellet of ~500 mg was obtained for each sample and DNA was extracted using the FastDNA™ Spin kit for Soil (Palex Medical, SA, Sant Cugat del Vallès, Barcelona, Spain) following the manufacturer’s instructions. DNA concentration and purity were determined by NanoDrop microspectrophotometry (ND-1000, Thermo Fisher Scientific, Waltham, MA, USA). 

#### 2.3.2. Sequencing Analysis

The Earth Microbiome Project pipeline (www.earthmicrobiome.org, 18 December 2018) was used for Illumina library preparation, sequencing, and core amplicon data analyses. The V4 region from the 16S rRNA using a 515F–806R primer pair was used for bacteria [29]. The ITS2 region using the primer pairs ITS1f-ITS2 was used for fungi [30]. PCR master mix and thermocycler conditions have been previously described [31]. The pooled sample was sequenced on the Illumina MiSeq platform using the Illumina MiSeq Reagent v3 600-cycle, at the Vincent J. Coates Genomics Sequencing Laboratory (UC Berkeley, Berkeley, CA, USA). 

#### 2.3.3. Data and Bioinformatic Analysis

QIIME2 version V2018.8 was used to analyze bacterial and fungal raw sequences. DADA2 was used for denoising and clustering (quality filtering) [32]. For fungal and bacteria analyses, UNITE (v7.2) and A 16S rRNA Greengenes database (http://greengenes.lbl.gov, 10 December 2018) were used. Thresholds of 96% and 100% were used for fungal and bacterial gene sequence identity, respectively. The obtained raw bacterial and fungal sequences associated with this study were deposited in the GenBank SRA database under BioProject accession number PRJNA780876. Statistical analysis was performed using the R Core Team (https://CRAN.R-project.org/package=here, 10 December 2018) with the vegan package.

### 2.4. Fungal and Bacterial Strains Isolation after Selective Pressure Experiment

#### 2.4.1. Isolation and Molecular Identification of Fungal and Bacterial Strains

After 9 weeks of selective pressure, a total of 6 flasks (3 from Kirk media and 3 from BH media) were used for the isolation of fungal and bacterial strains according to the methodology described by Waksman [33]. For each flask, 1 mL of content was mixed with 9 mL of sterilized saline solution (0.9% NaCl) and serial diluted. The dilutions were plated on Petri dishes containing the solid version of the same media (by the addition of 15 g/L of agar). In Kirk media, streptomycin and tetracycline were used as antibiotics (50 and 25 μg/mL) to avoid bacterial growth. Inoculated Petri dishes were incubated at 28 °C for fungi and 30 °C for bacteria for 15 days. All distinct morphotypes were isolated and subcultured on Malt Extract Agar (MEA) (28 °C) and Tryptic Soy Agar (TSA) (30 °C) for fungal and bacterial strains, respectively. The pure cultures were stored at room temperature and glycerol was prepared for long-term storage. 

Genomic DNA of fungal and bacterial isolated strains was extracted using the PrepMan^®^Ultra Kit following the manufacturer’s instructions. Then, a fresh colony was lysed in 100 mL of reactant and was heated at 100 °C for 10 min. Later, it was cooled down at room temperature for 2 min, centrifuged at 12,000 rpm for another 2 min, and finally 50 mL of the supernatant was transferred to a new tube to be stored at −20 °C. DNA obtained from isolates were amplified using pairs of primers. For fungi, ITS1 (5′-TCCGTAGGTGAACCTGCGG-3′) and ITS4 (5′-TCCTCCGCTTATTGATATGC-3′) [30] from the intragenic 5.8S region, and for bacteria, 27F (5′-AGAGTTTGATCMTGGCTCAG-3′) and 699R (5′-RGGTTGCGCTCGTT-3′ [34] to amplify 16S rRNA according to the conditions previously described [24]. Nucleotide sequences were submitted to the online BLAST search engine (http://www.ncbi.nlm.nih.gov/BLAST, accessed on 17 July 2020) of the National Centre for Biotechnology Information (NCBI). 

#### 2.4.2. Biodegradation Experiments of the Isolated Microorganisms

The isolated fungal and bacterial strains were individually tested to remove CBZ, KET and DCF. A fungal suspension for each strain was prepared and adjusted in a Neubauer chamber to inoculate 1 × 10^5^ spores/mL in 150 mL amber glass bottles containing 50 mL of the medium [35]. For bacterial strains, a pre-inoculum of each isolate was prepared in Tryptone Soy Broth (TSB). When maximum OD (λ = 600 nm) was reached, 2 mL of the culture was transferred into sterile Eppendorf tubes. These were then centrifuged at 14,000 rpm and washed with a sterile saline solution (0.85%), which was recovered in sterilized distilled water. Finally, 1 mL of each bacterial suspension was inoculated in 150 mL amber glass bottles containing 50 mL of BH medium. Flasks were cultured under agitation (120 rpm) at 28 °C and 30 °C for fungi and bacteria, respectively, for 48 h. After that, 100 μΜ of CBZ, KET and DCF was added to three systems: (a) active strain (biodegradation test), (b) inactivated strain by autoclaving (absorption control) (c) non-inoculated flask (abiotic control). Each determination was performed in triplicate. To analyze degradation kinetics, sacrificial flasks from each system were taken every seven days. Samples were centrifuged twice at 14,000 rpm for ten minutes and the collected supernatants were stored at −20 °C for HPLC analysis. 

### 2.5. Chromatographic Analyses 

#### 2.5.1. Characterization of Emerging Contaminants by LC/MS-QTOF in Sewage Sludge Samples

Lyophilized samples rSS and dSS (Section 2.1) were processed with a modified QuEChERS (Quick, Easy, Cheap, Effective, Rugged and Safe) extraction method [36]. One g of each sample was transferred into a 15 mL polypropylene tube. Following that, 2.2 mL of EDTA-McIlvaine buffer was added to the mixture that was shaken in a vortex for 1 min for homogenization. Subsequently, 5 mL of acetonitrile were added and then shaken for 1 min. After that, 1.25 g of (NH4)_2_SO_4_ was added to this mixture, which was vortexed for 1 min and centrifuged at 9000 rpm for 5 min. To continue, 5.5 mL of the supernatant were transferred into polypropylene tubes with 50 mg of C18, for the clean-up step, shaken again in vortexed for 1 min and centrifuged for 5 min at 5000 rpm. Finally, 5 mL of the supernatant were transferred into vials and dried with a gentle N_2_ steam at 40 °C. Final residue was re-dissolved with 0.5 mL of H_2_O-MeOH (95:5, v/v), vortexed for 2 min, filtered through 0.20 µm nylon syringe filters and injected into the SCIEX X500R QTOF system. Chromatographic separation was achieved on an Hibar^®^ HR Purospher^®^ STAR RP-C18 column (100 mm × 2.1 mm i.d., 2 µm particle size, Merck) using a mobile phase consisting of 0.05% aqueous formic acid solution (solvent A) and MeOH (solvent B) at a flow rate of 0.5 mL min^−1^. The gradient profile was as follows: 0 min, 5% B; 2.5 min, 5% B; 4.0 min, 100% B; 4.5 min, 100% B; 5.0 min, 5% B; 6.0 min, 5% B. The temperature of the column was 30 °C and the injection volume was 5 µL. A Sequential Window Acquisition of All Theoretical fragment ionic spectra (SWATH) was constructed as a second degree of data quantification at the Institute for Water Research Foundation of Catalonia (IDEA-ICRA). Samples were analyzed in triplicate.

#### 2.5.2. HPLC Analyses of the Removal of PhACs during Pressure Experiment and Biodegradation Experiments

An Agilent^®^ 1050 HPLC system (Waldbronn, Germany) was used to determine the efficiency in removing CBZ, KET and DCF in selective pressure experiments (Section 2.2.2) and in degradation experiments by microbial isolates (Section 2.4.2). This system was provided with a diode array detector (DAD; 190–700 nm) and a Synergi Fusion RP C18 column (4 μm, 4.6 Å~150 mm; Phenomenex^®^, Madrid, Spain). A volume of 10 μL of each sample was injected at a gradient flow rate of 0.9 mL/min, using a buffer of 85% of acetonitrile and 15% of H_2_O-H_3_PO_4_ (0.01%) for elution. Peak areas from determined absorbance at 278 nm were interpolated into a generated standard curve using 10–100 μM from each compound.

## 3. Results and Discussion

### 3.1. Occurrence of Emerging Pollutants in Sewage Sludge Samples 

**Figure 1:**
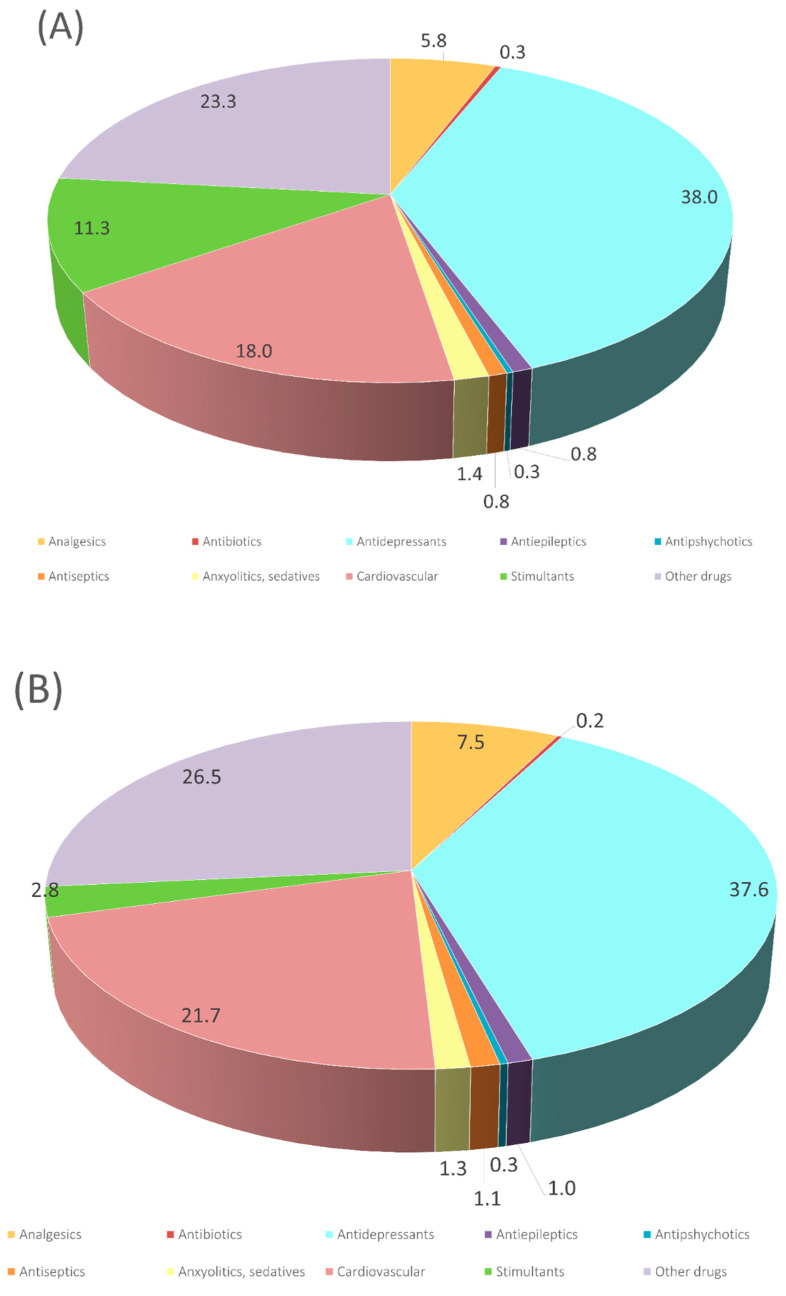
Summary of the different families of chemicals identified in (**A**) raw sewage sludge and (**B**) digested sewage sludge, with the normalized number of compounds per category.

The percentages found in rSS and dSS by LC/MS QTOF (raw and digested, respectively) are shown in Figure 1. After targeting 80 substances related to PhACs and potential candidates listed as priority substances [37] in raw and digested sludge, it was noticed that 34 compounds were present in one or both samples ranging from 1.0 to 1100 ng/g d.w. (Table 1). As shown in Figure 1, the profile of both sludges was very similar. The similar profile regarding the concentration of these compounds may indicate the inefficiency of anaerobic digestion under our conditions in removing pharmaceutical compounds. Indeed, there were a few compounds where the concentration of the content increased 70% more after digestion than before digestion (Table 1). These are: amlodipine, 1,2,3-benzotriazole, dexamethasone, diclofenac, fenofibrate, fluoxetine, metoprolol paroxetine and valsartan, whereas the concentration of acridone, atenolol, diltiazem, estrone, loratadine, mephedrone, valsartan acid and venlafaxine were reduced by more than 80% in the dSS. The most abundant compounds were sertraline (rSS 1100.0 ng/g d.w., dSS 864.4 ng/g d.w.), mephedrone (rSS 313.0 ng/g d.w., dSS 18.1 ng/g d.w.) and 1,2,3-benzotriazole (rSS 448.8 ng/g d.w., dSS 773.9 ng/g d.w.). Only three of the analyzed compounds were not detected (benzafibrate, citalopram and trimethoprim). The determination of PhACs will depend on different factors, such as: sampling strategy, precipitation levels, dilution of wastewater discharges, degradation within the sewer system [38], frequent industrial/agriculture activities, social trends [39,40,41,42] and population health condition [43,44]. A review of ECs content in wastewaters and raw sludge [45] showed rather different results in concentration ranges of ECs in wastewater influents, reporting higher concentrations for diclofenac (1500 ng/g d.w.), ketoprofen (102 ng/g d.w.) and carbamazepine (2593 ng/g d.w.) and were almost absent in raw sludge; only diclofenac was detected at 70 ng/kg in this matrix. Nevertheless, it is important to note that the lengthy chemical process of sludge digestion (20–30 days) does not help in the degradation of ECs, as most of the reported values stood at a similar range. Thus, after the digestion and storage of sewage sludge, some ECs will still persist [46]. More recently, the increase in the retention time of the anaerobic bioreactor or the two-phase process seems to be an efficient strategy in removing several PhACs [47].

### 3.2. Shift of Microbial Population during Selective Pressure Experiments

**Figure 2:**
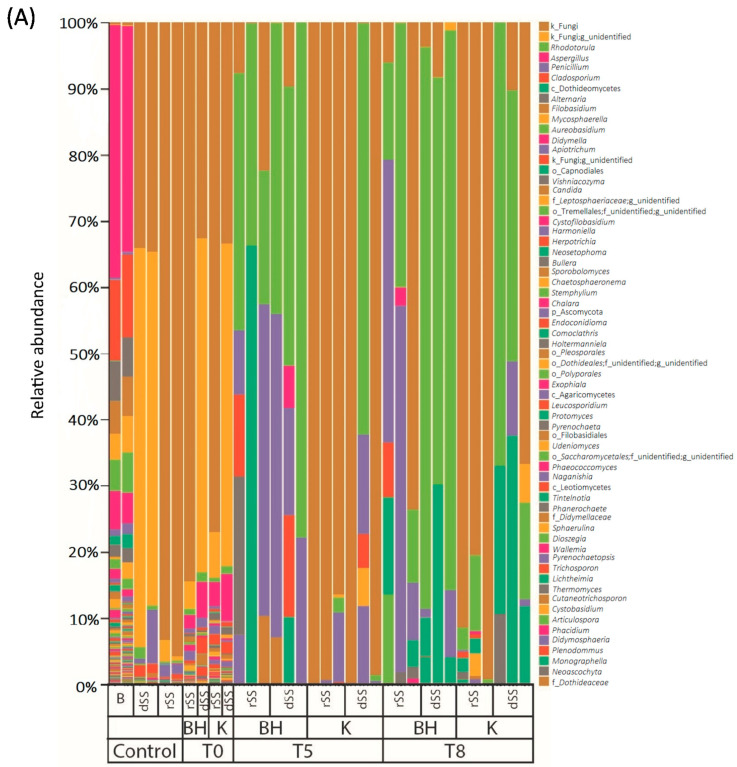

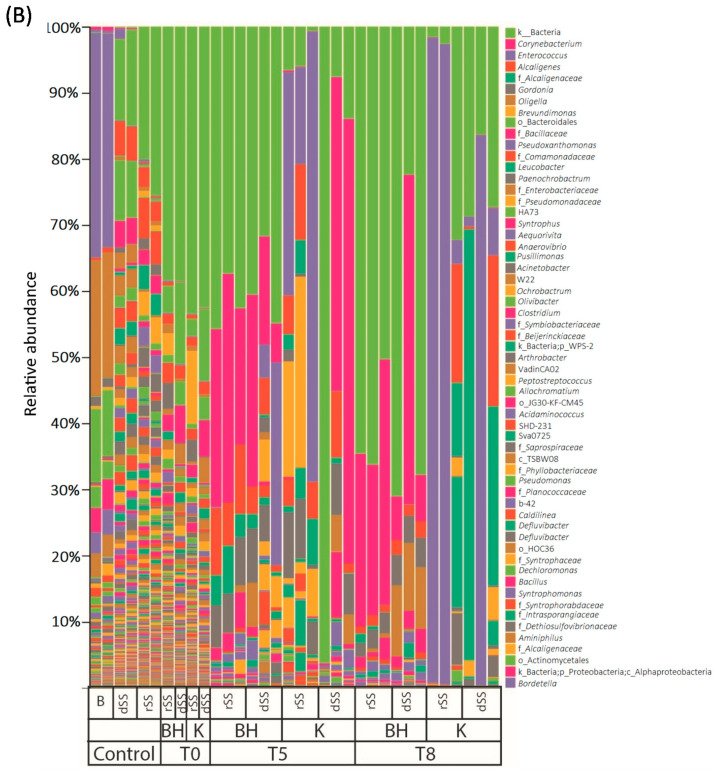
Relative abundance (%) in different media over time in the selective pressure experiments: (**A**) Fungal diversity and (**B**) bacterial diversity. B: Bulking, dSS: Digested Sewage Sludge, rSS: Raw Sewage Sludge, BH: Bushnell Hass Medium, K: Kirk medium, T: Time in weeks.

The analysis of the microbial communities was performed in the selective pressure experiment at weeks 0, 5 and 8 (Figure 2, Tables S3 and S4). The details of the sequence processing during the bioinformatics pipeline are shown in the SMT1 and SMT2. Regarding the fungal community, differences in the diversity between rSS and dSS were not found due to a high percentage of unidentified sequences. Representatives of the genera *Rhodotorula* and *Penicillium* were present in the dSS (more than 1%) and the genera *Apiotrichum* in the case of rSS (more than 1%). At the end of the experiment, *Rhodotorula* was the predominant genus in BH medium and dSS. Its abundance at the end of the experiment may indicate the potential of this genus to use pharmaceutical compounds as a carbon source. The percentage of *Rhodotorula* at the end of the experiment was higher than 60%. In the case of dSS, the abundance of *Rhodotorula* was higher compared to rSS. The rSS sample in the BH medium was more diverse than in the one cultivated in the Kirk medium. It contained genus such as *Penicillium*, *Stemphylium*, *Cladosporium*, *Aspergillus*, *Alternaria*, but also *Rhodotorula* (Figure 2A). The highest bacterial diversity was found in the starting material at the start of the experiment. After 5 weeks, the relative abundance showed a selection of microbial communities. Bacterial diversity in the dSS and rSS was very similar at the beginning of the experiment to that of the selective experiment, after the addition of the pharmaceutical compounds. The most abundant order was *Clostridiales* in rSS (more than 20% of abundance); *Bacteroidales* and *Synergistales* in dSS (more than 30% between both) (Figure 2B). Similar bacterial groups were detected in sludges after mesophilic anaerobic digestion, where the phylum *Bacteroidetes*, *Chloroflexi*, *Firmicutes*, and *Proteobacteria* were predominant [47].

BH medium favored the proliferation of *Actinomycetales* and *Burkholderiales* in rSS. The most abundant orders in Kirk medium were *Lactobacillales*, *Caudobacteriales* and *Rhizobiales* for the rSS. However, the bacterial structure in both media for dSS was similar. The diversity of the bacterial community decreased over time. After 8 weeks of experiments, less diversity was found in both sludges. The changes in the community were more affected by the medium used. For instance, in Kirk medium the genera found at the end of the experiment were *Enterococcus*, *Alcaligenes* or members of the *Alcaligenaceae* family. A different trend was observed in BH medium where no glucose was provided and *Corynebacterium* and *Oligella* were the most representative genera after 8 weeks of the experiment. This suggests that these genera could be more tolerant to the provided concentration of PhACs and, in the case of BH medium, be able to grow without any glucose addition, apart from the carbon source provided by the starting material. Therefore, both communities were different from the beginning and were selective according to the medium used.

### 3.3. Effect of the Sewage Sludge and the Medium for the Selection of Microorganisms 

**Figure 3:**
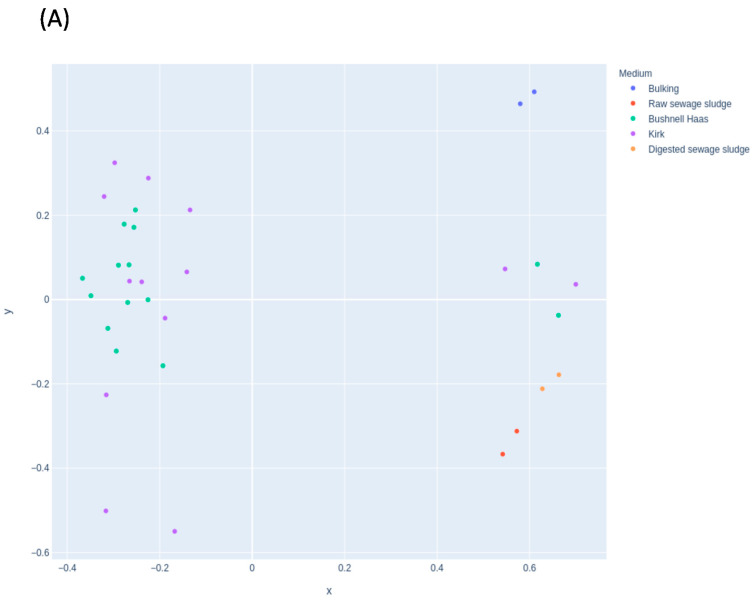

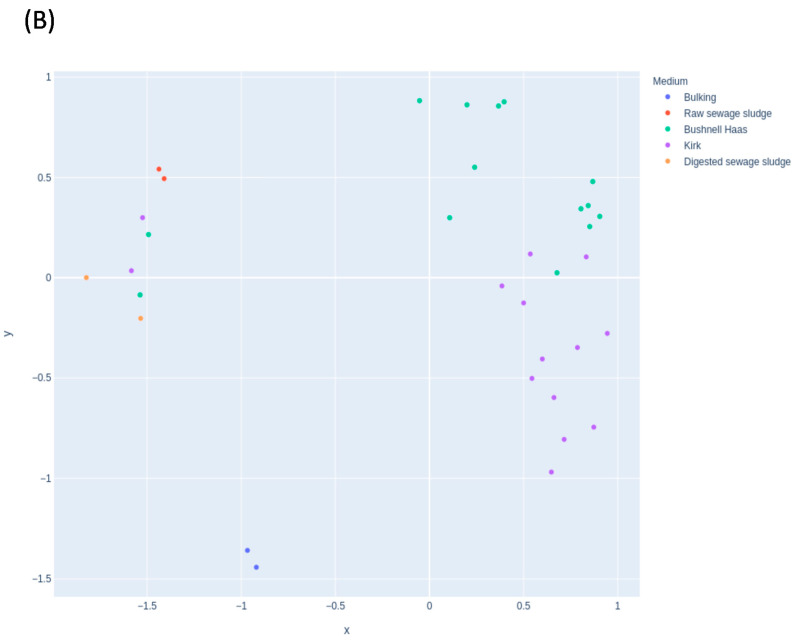
Multidimensional scaling (MDS) plot showing the distribution of samples in the enrichment experiment according to the medium used for (**A**) fungal and (**B**) bacterial communities.

Figure 3 shows the effect of the selected medium in the microbial communities. The MDS for fungal (Figure 3A) and bacterial (Figure 3B) communities did not show changes according to the medium at the starting condition used. Therefore, the use of BH or Kirk media did not affect the microbial communities that are grouped in the same clusters in Figure 3A. Time was the main factor that influenced the communities. The ones most affected were those that were contained in the starting material. However, in the case of bacterial communities, the use of different media shows different clusters (Figure 3B). This was also influenced by time. The bacterial community in Kirk medium at 5 weeks was similar to the BH media at 8 weeks. This may indicate the absence of a carbon source (such as in BH medium), which could stimulate the bacterial community with degradation abilities faster than in the presence of an extra carbon source (Kirk medium). 

### 3.4. Pharmaceutical Active Compounds (PhACs) Removal during Selective Pressure Experiment

**Figure 4:**
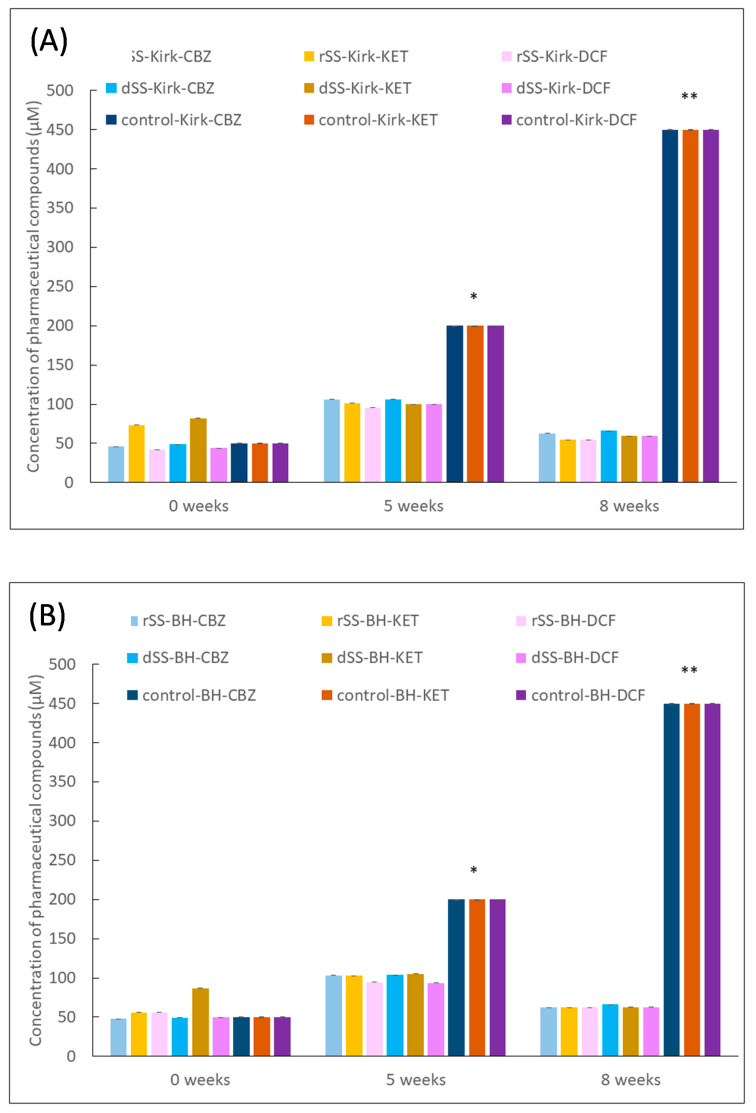
Overall concentration of PhACs (μM) after eight weeks of enrichment experiment with raw (rSS) and digested sewage sludge (dSS) in (**A**) modified Kirk medium and (**B**) BH medium. Mean values of triplicate measurements were calculated, 1 bar = standard deviation (n = 3). Number of asterisks (*, **) indicated significant differences *p* < 0.05 calculated by three-way ANOVA (Sigmaplot v.12.0).

The removal of PhACs by the autochthonous microbiota in the selective pressure experiment is shown in Figure 4. The figures display the concentration of pharmaceutical compounds CBZ, KET and DCF detected during the eighth week of selective pressure. These results show a concentration range of 50–110 μM for each compound, indicating no accumulative effect over time, as well as the presence of microorganisms’ abilities to remove the added compounds. The concentration of PhACs increased after 5 weeks, as was expected, since every week the same concentration of PhACs was added. This suggests that microbial activity partially degraded these three compounds, since these were not accumulated after 8 weeks. In contrast, the concentration of PhACs in control treatments increased over time due to the absence of microbial activity. It also indicates a positive effect in the degradation by native microorganisms in both sludges (rSS and dSS). In a similar way, Baratpour and Moussavi [48] used a 90-day selective pressure experiment of a bacterial biofilm (a mixture of *Pseudomonas* spp. and *Bacillus* spp.) with 100 mg/L of acetaminophen (ACT) for biomass acclimation and 6 mM of H_2_O_2_ as a stimulation for accelerating the removal efficiency, completely removing ACT on a fixed bed reactor every 10 days. Another approach of a three-step enrichment procedure was made by Iranzo et al. [18], in which a compost made of WWTP sludge and rice straw was suspended in Yeast Extract Peptone Dextrose (YPD) and a complete minimal medium with and without a carbon and nitrogen source to aim for the best outcome of biodegrading. PhACs such as azithromycin, benzylpenicillin, citalopram, fluconazole, fluoxetine, ibuprofen, irbesartan, olanzapine, telmisartan, and venlafaxine were best degraded at a C/N ratio of 20. Azithromycin levels were reduced by up to 50%, citalopram was reduced by 10%, and fluoxetine was completely biodegraded over 15 days of treatment.

### 3.5. Isolation of Microorganisms after Selective Pressure

After 63 days of selective pressure with CBZ, KET and DCF, the isolation of fungal and bacterial strains was performed. At the end of the experiment, a total of 7 fungi and 11 bacteria were isolated and identified through the amplification of the ITS and 16S rRNA genes. Table 2 shows fungi and bacteria isolates. The obtained sequences of fungal and bacterial isolates associated with this study were deposited in the GenBank database under the accession number (Table 2). Fungal species found were: *Cladosporium cladosporioides*, *Cladosporium limoniforme*, *Cladosporium halotolerans*, *Alternaria alternata*, *Aspergillus montevidensis*, *Penicillium raistrickii* and *Purpureocillium lilacinum*. All of them belong to the *Ascomycota* phylum, the biggest taxonomic group of the fungal kingdom. This includes industrial, medical and biological model species and also the highest percentage found by non-culture techniques. These isolated fungal strains found at the end of the experiment were the most representative found by non-culture techniques. 

Bacterial species identified were *Bacillus simplex*, *Corynebacterium efficiens*, *Corynebacterium humireducens*, *Gordonia hirsute*, *Alcaligenes faecalis*, *Micrococcus yunnanensis*, *Enterococcus faecium*, *Paenalcaligenes hominis*, *Oligella ureolytica*, *Sphingobacterium jejuense* and *Staphylococcus hominis*. These showed a mixture of different phyla such as Actinobacteria and Proteobacteria (Table 2). These co-existing bacteria had rarely been reported as xenobiotic degraders, whereas other bacteria belonging to the same phylum had remarkable results with a contingency of PhACs. One example of this capability was described by Thelusmond et al. [49], showing a partial degradation of CBZ, triclocarban and triclosan in soil after 80 days of biostimulation with native microorganisms. Illumina amplicon sequencing results showed that *Rhodococcus* sp., *Streptomyces* sp. (both Actinobacteria), *Pseudomonas* sp., *Sphingomonas* sp. and *Methylobacillus* sp. (the last three are Proteobacteria) were the most abundant during the process. The adaptation and tolerance of these isolates to a stable concentration of PhACs open the possibility of exploring metabolic profiles in order to perform enzyme extraction, as has been previously suggested [50,51]. 

### 3.6. Biodegradation Experiments Using the Isolated Strains

**Figure 5:**
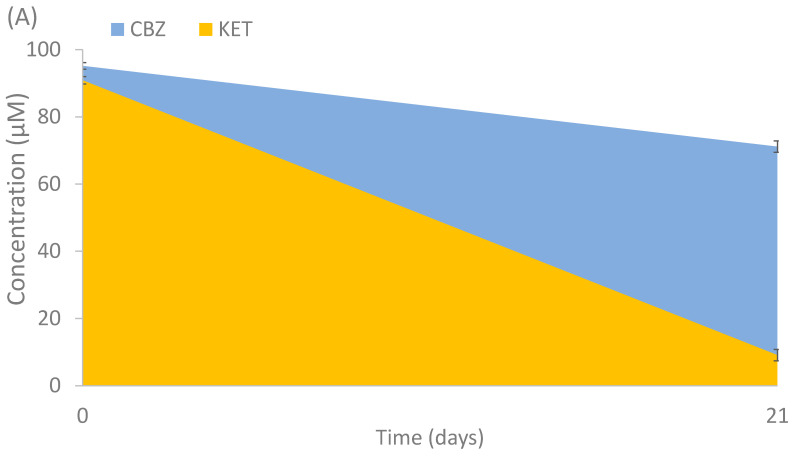

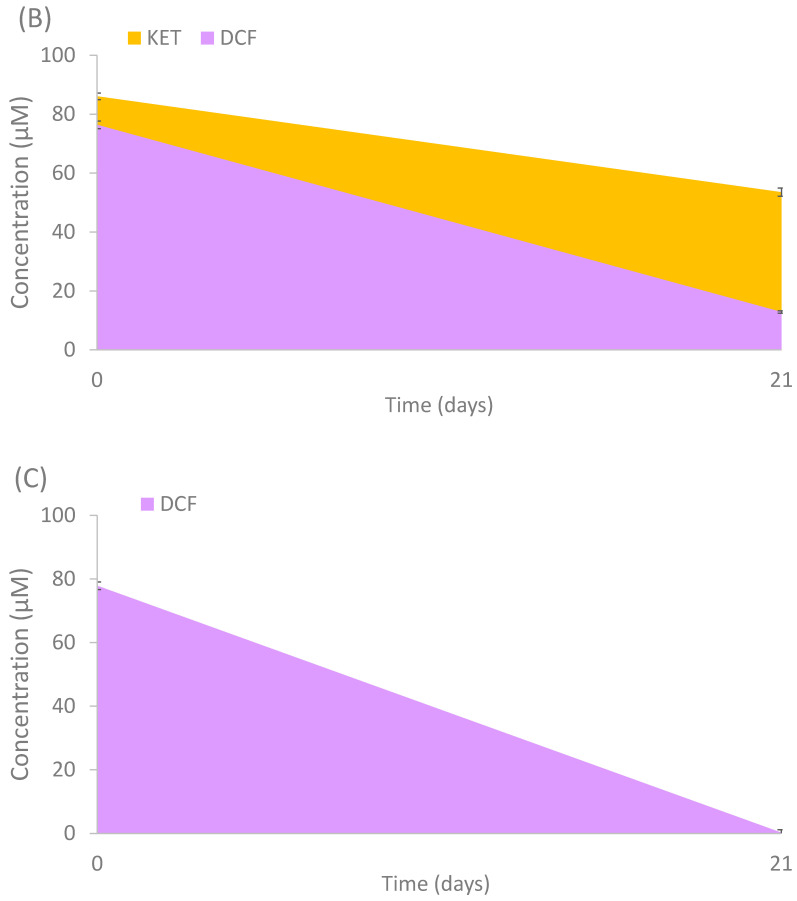
Degradation of selected PhACs by (**A**) *Cladosporium cladosporioides*, (**B**) *Alternaria alternata* and (**C**) *Penicillium raistrickii*. Mean values of triplicate measurements were calculated, and error bars represent standard deviation (n = 3).

To evaluate the capacity of the isolated strains to degrade the studied compounds, and in order to select potential candidates for consortia degradation, a biodegradation experiment was performed with the isolates. The potential of degradation by the total number of isolates (18) were evaluated individually. Only 3 out of 18 fungal strains showed positive results. *Cladosporium cladosporioides*, *Alternaria alternata* and *Penicillium raistrickii* showed partial and total degradation activity after 21 days of incubation (Figure 5). CBZ had a minor reduction (25.2%) by *C. cladosporioides*, KET was mostly consumed (90%) by the same fungal isolate (Figure 5A) and partially degraded (37.8%) by *Alternaria alternata*; DCF was almost totally consumed by *Alternaria alternata* (83.1%) (Figure 5B) and *Penicillium raistrickii* (99.6%) (Figure 5C). The use of fungi previously isolated from polluted environments has been studied for the removal of PhACs, such as those performed by Conejo-Saucedo et al. [35], in which *Talaromyces gossypii*, *T. verruculosus*, *Aspergillus terreus*, *A. cejpii* and *Syncephalastrum* *monosporum* were able to degrade between 14.6 and 84.6% of DCF in 3 days. The main mechanisms in degrading these compounds are the intra and extracellular enzymes and/or sorption processes on fungal biomass. For instance, *Trametes versicolor* and *Gymnopilus luteofolius* have shown a good degradation of carbamazepine (90 and 95%, respectively), which is considered a very recalcitrant compound [52]. *Bjerkandera adusta* has shown a removal efficiency of 100% for diclofenac using versatile peroxidase [53]. The effectiveness of removal found by the strain *P. raistrickii* (99.6%) for diclofenac and by *C. cladosporioides* for ketoprofen (90%) is higher than the degradation of *T. versicolor* for the same compounds (95 and 48%, respectively) [54]. To our knowledge, enrichment experiments with PhACs have not been carried out using fungi. They allow us to find species that are not often studied in the degradation of PhACs which have a high potential of degradation. In some cases, these may be used as good candidates for bioreactor strategies amongst others. For instance, *Phanerochaete chrysosporium* isolated from polluted sites removed between 50 and 60% of diclofenac and other NSAIDS in a bioreactor after 100 days (5 mg L^−1^) [55]. Other studies with fungi have shown better degradation in immobilized systems than in suspensions systems [56]. This is similar to the degradation percentage caused by *T. versicolor*, other systems such as bio-slurry, and solid phase systems using exogenous fungi. In this case, the application of this white-rot fungus, which assisted the biodegradation experiment of CBZ and naproxen, achieved partial degradation (57 and 47%, respectively) after 24 h [57]. 

## 4. Conclusions

Sewage sludge represents a significant source of PhACs and adapted microbial populations. Selective pressure systems allow us to obtain and elucidate the most tolerant microbial communities and identify those microorganisms with potential abilities to degrade emerging pollutants. Despite further studies being needed, pressure selective systems may be considered a first step to implant bioaugmentation strategies in sewage sludge composting and deal with the problem of emerging pollutants. Further experiments should focus on studying the degradation potential of emerging pollutants by culturable consortia in order to use them as an integral biodegradation strategy.

## Figures and Tables

**Table 1:** Pharmaceutical active compounds content expressed as ng/dw found in Sewage sludge (raw and digested sewage sludge) using LC/MS/QTOF.

Compound	Formula	CAS Number	Application	rSS (ng/g, d.w.)	dSS (ng/g, d.w.)	RSD (%)	Percentage of Removal
Acetaminophen	C_8_H_9_NO_2_	103-90-2	Analgesic	45.3	31.8	20.3	29.9
Acridone	C_13_H_9_NO	578-95-0	Antiviral agent	3.9	0.3	13.0	91.3
Amlodipine	C_20_H_25_ClN_2_O_5_	88150-42-9	Cardiovascular	BLOQ	148.3	6.4	
Atenolol	C_14_H_22_N_2_O_3_	29122-68-7		48.7	0		100.0
1.2.3-Benzotriazole	C_6_H_5_N_3_	95-14-7	Drug precursor	448.9	773.9	13.0	−72.4
Caffeine	C_8_H_10_N_4_O_2_	58-08-2	Stimulant	BLOQ	37.6	43.2	
Carbamazepine	C_15_H_12_N_2_O	298-46-4	Antiepileptic	8.7	6.7	4.8	23.6
Carbamazepine-10.11-epoxide	C_15_H_12_N_2_O_2_	36507-30-9	Carbamazepine metabolite	13.5	16.8	5.3	−24.4
Chlorpromazine	C_17_H_19_ClN_2_S	50-53-3	Antipsychotics	8.4	9.3	14.3	−11.0
Dexamethasone	C_22_H_29_FO_5_	50-02-2	Analgesic (corticosteroids)	117.8	148.7	29.3	−26.2
Diclofenac	C_14_H_11_Cl_2_NO_2_	15307-86-5	Analgesic	17.3	38.9	16.4	−124.7
Diltiazem	C_22_H_26_N_2_O_4_S	42399-41-7	Cardiovascular	17.5	3.1	9.6	82.0
Estrone	C_18_H_22_O_2_	53-16-7	Hormone	302.7	18.1	25.7	94.0
Fenofibrate	C_20_H_21_ClO_4_	49562-28-9	Cardiovascular	142.4	318.5	35.2	−123.7
Fluoxetine	C_17_H_18_F_3_NO	54910-89-3	Antidepressant	57.5	135.9	1.8	−136.4
Ketamine	C_13_H_16_ClNO	6740-88-1	Anesthetic	1.0	0.6	41.9	37.6
Ketoprofen	C_16_H_14_O_3_	22071-15-4	Analgesic	10.0	5.7	11.1	42.6
Lamotrigine	C_9_H_7_Cl_2_N_5_	84057-84-1	Antiepileptic.	4.9	5.7	9.7	−17.0
Loratadine	C_22_H_23_ClN_2_O_2_	79794-75-5	Antihistamine	4.2	0		100.0
Lormetazepam	C_16_H_12_Cl_2_N_2_O_2_	848-75-9	Anxiolytic, sedative	9.8	12.0	8.0	−22.7
Mephedrone	C_11_H_15_NO	1189805-46-6	Stimulant drug	313.0	18.1	6.4	94.2
Methadone	C_21_H_27_NO	76-99-3	Stimulant drug	54.6	24.4	24.0	55.3
Metoprolol	C_15_H_25_NO_3_	37350-58-6	Cardiovascular	11.5	21.7	15.6	−88.9
Oxazepam	C_15_H_11_N_2_O_2_Cl	604-75-1	Anxiolytic	20.7	17.7	6.3	14.8
Paroxetine	C_19_H_20_FNO_3_	61869-08-7	Antidepressant	54.1	121.9	17.5	−125.2
Propranolol	C_16_H_21_NO_2_	525-66-6	Cardiovascular	33.5	44.1	17.2	−31.7
Sertraline	C_17_H_17_Cl_2_N	79617-96-2	Antidepressant	1100.0	864.4	3.5	21.4
Sulfapyridine	C_11_H_11_N_3_O_2_S	144-83-2	Antibiotic	10.9	7.5	18.9	31.7
Temazepam	C_16_H_13_ClN_2_O_2_	846-50-4	Anxiolytic, sedative	10.4	4.9	5.9	52.4
Triclocarban	C_13_H_9_Cl_3_N_2_O	101-20-2	Antiseptic	24.6	34.1	21.7	−38.5
Valsartan	C_24_H_29_N_5_O_3_	137862-53-4	Cardiovascular	58.2	112.3	8.8	−93.0
Valsartan acid	C_14_H_10_N_4_O_2_		Valsartan metabolite	275.5	0		100.0
Venlafaxine	C_17_H_27_NO_2_	93413-69-5	Antidepressant	30.8	2.8	7.0	91.0
Zolpidem	C_19_H_21_N_3_O	82626-48-0	Insomnia treatment	5.3	5.4	19.8	−2.0

**Table 2:** Identified isolated microorganisms according to the 99% of database similarity, Gene bank ascension number.

	Isolate ID	>99% Database Similarity	Gene Bank Accession Number	Phylum
Fungal strains	H1	*Cladosporium cladosporioides*	MT773569	Ascomycota
	H2	*Cladosporium limoniforme*	MT773579	Ascomycota
	H3	*Cladosporium halotolerans*	MT773604	Ascomycota
	H4	*Alternaria alternata*	MT776719	Ascomycota
	H5	*Aspergillus montevidensis*		Ascomycota
	H6	*Penicillium raistrickii*		Ascomycota
	H7	*Purpureocillium lilacinum*	MT773618	Ascomycota
Bacterial strains	C1	*Bacillus simplex*	MT773382	Firmicutes
	C2	*Corynebacterium efficiens*	MT773417	Actinobacteria
	C3	*Corynebacterium humireducens*	MT773419	Actinobacteria
	C4	*Gordonia hirsuta*	MT773437	Actinobacteria
	M1	*Alcaligenes faecalis*	MT773443	Proteobacteria
	K1	*Micrococcus yunnanensis*	MT773451	Actinobacteria
	K4	*Enterococcus faecium*	MT773438	Firmicutes
	T1	*Paenalcaligenes hominis*	MT773452	Proteobacteria
	T4	*Oligella ureolytica*	MT773453	Proteobacteria
	T15	*Sphingobacterium jejuense*	MT773454	Bacteroidetes
	T19	*Staphylococcus hominis*	MT773567	Firmicutes

## Supplementary Materials

The following supporting information can be downloaded at: https://www.mdpi.com/article/10.3390/jof8070668/s1, Figure S1: Workflow of the selective pressure experiment set-up; Table S1: Composition of modified Kirk medium used in the selective pressure experiment Table S2: Composition of BH medium used in the selective pressure experiment. Table S3: Summary metric of bacterial community analyses in the bioinformatic pipeline. Table S4: Summary metric of bacterial community analyses in the bioinformatic pipeline.
