# Supplementary material for: High-Throughput Microbial Community Analyses to Establish a Natural Fungal and Bacterial Consortium from Sewage Sludge Enriched with Three Pharmaceutical Compounds
Source: J Fungi (Basel). 2022 Jun 25;8(7):668. doi: 10.3390/jof8070668 (PMC9324927; doi:10.3390/jof8070668)
Supplement: Supplementary file 1 [file jof-08-00668-s001.zip › jof-1777148-supplementary.pdf]

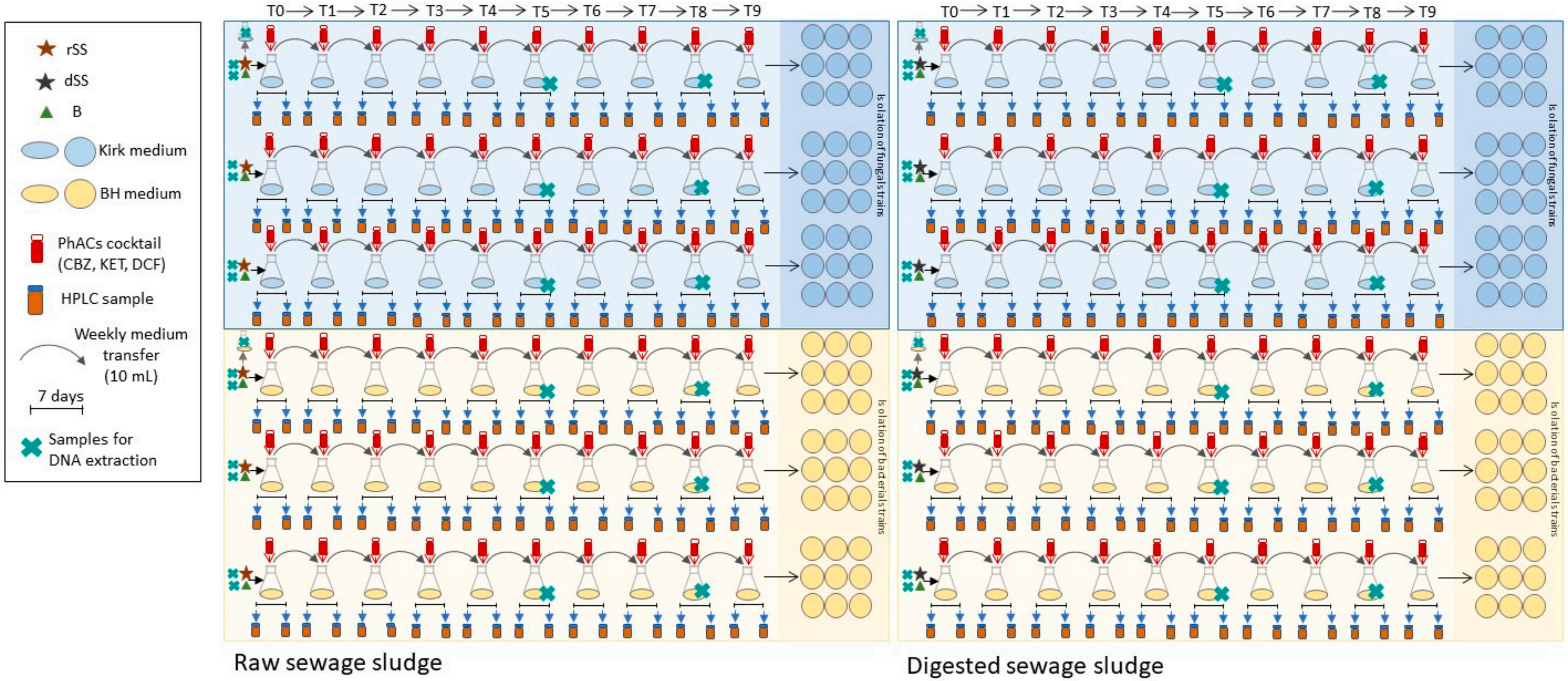

Figure S1. Workflow of the selective pressure experiment set-up. Brown stars represent raw sewage sludge (rSS), dark grey stars represent digested sewage sludge (dSS), green triangle represent bulking agent (B). Arrows shown the transfer and small vials represent when the PhACs were added (dark red vial) and when the samples for HPLC were taken (blue cap vials). The time-scale is represented with a straight line between flasks and corresponds to 1 week. Blue crosses indicate when samples were taken for DNA and sequencing analysis. Light blue represents those flasks where modified Kirk medium was used and light yellow for BH medium. Isolation was performed in the final flask in the appropriate media.

Table S1. Composition of modified Kirk medium used in the selective pressure experiment.

Compound	g/L
Glucose	5
Yeast extract	1
Peptone	2
Ammonium tartrate	2
KH_2PO_4	0.2
$MgSO_4 \cdot 7H_2O$	0,5
KCl	0,5
Mineral solution	1 (mL)

Mineral solution	g/L
$B_4O_7Na_2 \cdot 10H_2O$	0.1 g
$CuSO_4 \cdot 5H_2O$	0.01
$FeSO_4 \cdot 7H_2O$	0.05
$MnSO_4 \cdot 7H_2O$	0.01
$ZnSO_4 \cdot 7H_2O$	0.07
$(NH_4)_6Mo_7O_{24}$ $4H_2O$	0.01

Table S2. Composition of BH medium used in the selective pressure experiment.

Compound	g/L
$MgSO_4$	0.2
$CaCl_2$	0.02
KH_2PO_4	1
K_2HPO_4	1
$(NH_4)_2SO_4$	1
$FeCl_3$	0.05

Table S3. Summary metric of fungal community in the bioinformatic pipeline.

Table S3.1 Summary metric of fungal community analyses.

Metric	Sample
Number of samples	34
Number of features	1,449
Total frequency	1,443,807

Table S3.2. Frequency per sample.

**Frequency per Sample**	
Minimum frequency	880.0
1st quartile	5,941.75
Median frequency	42,332.0
3rd quartile	58,658.75
Maximum frequency	147,374.0

Mean frequency	42,464.91176470588

Table S3.3 Frequency per feature

**Frequency per feature**	
	Frequency
Minimum frequency	1.0
1st quartile	32.0
Median frequency	88.0
3rd quartile	274.0
Maximum frequency	71,053.0
Mean frequency	996.416149068323

Table S3.4. Ratios (%) of sequences retained after the denoising process using DADA2 for the pipeline flows applied to the data.

	**Demultiplexed**	**DADA2**	
	**Number**	**Number**	**% Retained**
**Samples**	11	11	100.00
**Total sequences**	1866191	318247	17,05
**Av seq/sample**	169654	28931	17,05
**Max sequences**	240058	60086	25,03
**Min sequences**	93640	41567	44,39
**Total features**	-	290	100

Table S4. Summary metric of bacterial community analyses in the bioinformatic pipeline.

Table S4.1. Frequency per Sample.

**Metric**	**Sample**
Number of samples	34
Number of features	5,114
Total frequency	3,875,414

Table S4.2. Frequency per sample.

**Frequency per Sample**	
Minimum frequency	11,629.0
1st quartile	68,015.0
Median frequency	108,523.0
3rd quartile	150,170.25
Maximum frequency	248,936.0
Mean frequency	113,982.76470588235

Table S4.3 Frequency per feature

**Frequency per feature**	
	Frequency

Minimum frequency	1.0
1st quartile	14.0
Median frequency	48.0
3rd quartile	176.0
Maximum frequency	379,462.0
Mean frequency	757.8048494329292

Table S4.4. Ratios (%) of sequences retained after the denoising process using DADA2 for the pipeline flows applied to the data.

	Demultiplexed	DADA2	
	**Number**	**Number**	**% Retained**
**Samples**	11	11	100.0
**Total sequences**	1735722	1197039	68,96
**Av seq/sample**	157793	108822	68,96
**Max sequences**	240058	147641	61,50
**Min sequences**	93642	76201	81,37
**Total features**	-	806	100
